# Macrophage migration inhibitory factor (rs755622) gene polymorphism in vitiligo

**DOI:** 10.1007/s11845-025-03966-9

**Published:** 2025-05-30

**Authors:** Doaa Falih Hadi, Abdel-Aziz Ibrahim El-Taweel, Amany Ibrahim Mustafa, Ola Samir El-Shimi, Mahmoud A. Rageh

**Affiliations:** 1 Department of Dermatology, Faculty of Medicine, Benha University, Benha, Egypt; 2 Department of Clinical and Chemical Pathology, Faculty of Medicine, Benha University, Benha, Egypt; 3 Department of Dermatology, Faculty of Medicine, Al-Azhar University, Cairo, Egypt

**Keywords:** Autoimmune diseases, Macrophage migration inhibitory factor, Polymorphism genetics, Vitiligo

## Abstract

**Background:**

Vitiligo is a cutaneous disease caused by the destruction of functioning melanocytes. Cellular immunity is known to have a role in the pathogenesis of vitiligo. Macrophage migration inhibitory factor (MIF) is a potent activator of macrophages and is considered to play an important role in cell-mediated immunity.

**Aims:**

We tried to investigate the association between MIF (rs755622) gene polymorphism and vitiligo susceptibility and its relationship to severity and clinical types of the disease.

**Methods:**

Fifty patients with vitiligo and 50 healthy controls were included. Determination of MIF gene (rs755622) single nucleotide polymorphism was conducted using polymerase chain reaction (PCR).

**Results:**

There were 18 males and 32 females who had vitiligo, together with 50 control participants of matched age and gender. Taking GG genotype and G allele as references, MIF GC, CC, (GC + CC) genotypes, and C allele demonstrated a substantially higher occurrence in all patients when compared to controls, with a higher risk of developing vitiligo within healthy control subjects.

**Conclusions:**

To the best of our knowledge, this is the third study worldwide and the first from Egypt to investigate the association between MIF gene polymorphism (rs755622) with vitiligo susceptibility and severity, and it showed that MIF gene polymorphism (rs755622) may be considered a risk factor for vitiligo susceptibility. Moreover, it may be associated with higher disease extent.

## Introduction

Vitiligo is a pigmentary disorder marked by the development of depigmented macules and patches caused by the progressive loss of epidermal melanocytes. It has a global incidence of 0.5–2%, with 50% of cases commencing before the age of 20. Although vitiligo susceptibility is not thought to be sex-related, 6–38% of patients have family members who have the condition, indicating a hereditary connection. However, the disorder’s inheritance pattern is compatible with a polygenic feature rather than transmission via a simple Mendelian basis [1, 2].

Etiopathogenesis of vitiligo is multifactorial, consisting of immunological, neurogenic, genetic, and environmental factors [3]. Studies strongly imply that different genetic polymorphisms are involved in the pathogenesis of vitiligo; also, changes in the immune system are responsible for the condition. The autoimmune theory proposes that alterations in humoral or cellular immunity result in the destruction of melanocytes [4].

Macrophage migration inhibitory factor (MIF), also known as glycosylation-inhibiting factor (GIF) is a protein that in humans is encoded by the MIF gene on chromosome 22q11.2 [5]. MIF plays a role in the regulation of macrophage function in host defense through the suppression of the anti-inflammatory effects of glucocorticoids [6]. MIF is an immune regulatory cytokine that is produced primarily by macrophages and T cells and conversely participates in their activation [7].

MIF gene encodes a lymphokine involved in cell-mediated immunity, immune regulation, and incorporated in various immune skin disorders like bullous pemphigoid, psoriasis, vitiligo, systemic sclerosis, lupus erythematosus, and atopic dermatitis [8, 9].

The aim of this study was to investigate the association between MIF (rs755622) gene polymorphism and vitiligo susceptibility and its relationship to severity and clinical types of the disease among the studied group of patients.

## Materials and methods

### Study design

This prospective case–control study was conducted at the Department of Dermatology, Faculty of Medicine, Benha University after approval by the Research Ethics Committee. The study was performed in accordance with the Declaration of Helsinki. Written consent was obtained from all participants after being informed about the nature of the study.

### Study population

There were 50 vitiligo patients enrolled in the study (Group A) [Different clinical variants and degrees of severity of vitiligo according to distribution and body surface area percentage (BSA%) affection]. In addition, as a control group (Group B), 50 healthy individuals of similar age and sex were included. Patients suffering from chronic diseases or other autoimmune disorders were excluded from the study.

All patients were subjected to a thorough history-taking process that included gathering information on their age, sex, drug use history, family history of vitiligo, age at onset, course and duration of the disease, history of Koebnerization, period of stability, previous treatments, and any chronic illnesses, thyroid conditions, or other autoimmune disorders. Clinical examination was done to evaluate the affected sites, the clinical type, and the extent of vitiligo (BSA%).

The BSA affected by vitiligo was calculated according to the rule of nines, where the surface area of the palm of the patient was considered to represent 1% of BSA. Body surface area is divided into areas each of which represents 9% (two upper limbs, head and neck, anterior and posterior surface of each lower limb). The anterior and posterior surfaces of the trunk each represent 18%. The genitalia and the perineum represent the remaining 1% [10]. The stability was evaluated by the vitiligo disease activity (VIDA) score (neither the presence of new lesions nor an increase of the old lesions for ≥ 1 year) [11].

### Molecular biology investigation

Determination of MIF (rs755622) gene single nucleotide polymorphism through polymerase chain reaction (PCR) in all studied subjects.

#### Sampling

From each participant, 3 ml of venous blood was aspirated and deposited on tubes containing EDTA (1.2 mg/ml) as an anticoagulant before preservation at − 20 °C for further molecular biology evaluation.

#### DNA extraction

From whole blood, DNA was extracted by QIAamp® DNA Blood Mini Kit (Cat# 51.104, QIAGEN, Germany) according to manufacturer protocol.

#### Principle

QIAGEN Protease, an enzyme which is completely free of DNase and RNase activity, is used to induce cell lysis to get the nucleic material out of the nuclear and cellular membranes. Then, the DNA purification procedure is carried out in four steps using QIAamp Mini spin columns in a standard micro-centrifuge by first allowing the DNA only in the lysate to be adsorbed to the QIAamp silica membrane. Then, DNA bound to the QIAamp membrane is washed in two centrifugation steps using two different wash buffers (Buffer AW1 and Buffer AW2) to ensure complete removal of any residual contaminants and to improve the purity of the eluted DNA in DNase/RNase free water.

#### Assessment of DNA quality and concentration

A NanoDrop2000 (Thermo Scientific, Wilmington, Germany) was used to assess the DNA quality at OD 260/280 against DNase/RNase-free sterile water as blank. The A260/A280 ratio between 1.8 and 2 was accepted as pure DNA. DNA concentration was measured with the same machine, and any sample with concentration < 10 ng/µl was excluded.

#### Genotyping of MIF (rs755622) gene polymorphism

Genotyping of MIF (rs755622) gene polymorphism was accomplished using TaqMan® SNP Genotyping Assay, done in accordance with the manufacturer’s instructions.

#### Performing PCR

PCR was used to enzymatically amplify the extracted DNA on a PikoReal 24 thermal cycler from Thermo Scientific.

#### Interpretation of results

The fluorescence released from the corresponding dye, which shows the alleles present in the sample, was used to determine the genotype of each sample. The genotyped data was organized for later statistical analysis and exported to Excel sheets.

### Statistical analysis

The collected data were revised, coded, tabulated, and introduced to a PC using Statistical Package for the Social Sciences (SPSS), Version 24.0 (IBM Corp., Armonk, New York, USA). Data were presented, and suitable analysis was done according to the type of data obtained for each parameter.

## Results

In addition to 50 healthy controls, the present study included 50 patients, 18 males (36%) and 32 females (64%), who suffered from vitiligo. Their mean age was 25.4 years. No significant differences were found in family history between all studied groups (Table 1).

**Table 1:** Comparison of age, sex, and family history in the studied groups

		Control *N* = 50		Vitiligo *N* = 50		*p*
Age (years)	Mean ± SD	27.4 ± 6.9		25.4 ± 7.3		0.553^*^
Male	*N*, %	22	44%	18	36%	0.414^**^
Female	*N*, %	28	56%	32	64%	
Positive family history		2	4	4	8	0.678^***^

*SD* standard deviation

^*^t test

^**^Chi square test

^***^Fisher exact test

Regarding the affected sites in all studied patients, face and scalp were affected in 22%, neck in 14%, trunk in 58%, elbows and knees in 24%, head and feet in 58%, forearm in 26%, and legs in 44%. Half of the patients showed vitiligo vulgaris, 26% had acral type, 8% had acrofacial, 14% had generalized type, and 2% had vitiligo universalis. Median BSA% affected was 4, ranged from 0.25 to 70, median age of onset was 12, ranged from 1 to 53 years. Four cases had a stationary course (8%), while 46 had a progressive course.

This sample of unrelated individuals was a random selection from inhabitants of Qalyubia Governorate in Delta Egypt. Applying Hardy–Weinberg (HW) equation showed that MIF genotypes in both groups were in HW equilibrium (Table 2).

**Table 2:** Assessment of Hardy–Weinberg equilibrium in the studied groups

	Control N = 50		Vitiligo N = 50	
	Observed	Expected	Observed	Expected
GG	38	37	20	20.5
GC	10	12	24	23
CC	2	1	6	6.5
p	0.231		0.786	

Deviations from Hardy–Weinberg equilibrium expectations were determined using the chi-square test

Taking GG genotype and G allele as references, MIF GC, CC, (GC + CC) genotypes, and C allele showed significantly higher frequency in all patients when compared to controls, with higher risk of developing vitiligo within healthy control subjects (*p* value 0.001) as shown in Table 3.

**Table 3:** Comparison of MIF genotypes and alleles distribution frequency between control group and vitiligo cases

		Control *n* = 50		Vitiligo *n* = 50		*p*	OR	95% CI
		N	%	N	%			
Genotypes	*GG*	38	76	20	40		1	(Reference)
	*GC*	10	20	24	48	0.001	2.562	1.471–4.462
	*CC*	2	4	6	12	0.035	2.927	1.076–7.962
	*GC* + *CC*	12	24	30	60	< 0.001	2.626	1.559–4.422
Alleles	*G*	86	86	64	64		1	(Reference)
	*C*	14	14	36	36	< 0.001	3.455	1.721–6.937

Logistic regression test was used

*OR* odds ratio, *CI* confidence interval

No significant differences were found in MIF genotype distribution regarding age or gender in the studied vitiligo group.

Percentage of affected surface area among cases varied significantly regarding genotypes, and the highest BSA% was associated with the CC genotype (Table 4).

**Table 4:** Comparison of genotype distribution according to BSA%, onset, and course in vitiligo group

Vitiligo		*GG*	*GC*	*CC*	*GC* + *CC*	*P1*	*P2*
		*N* = 20	*N* = 24	*N* = 6	*N* = 30		
**BSA%**	**Median**	**1**	**4.5**	**50**	**8**	< 0.001^*^	0.002^**^
	**Range**	**0.25–25**	**0.25–50**	**40–70**	**0.25–70**		
**Age of onset**	**Median**	**11.5**	**14**	**20.5**	**14**	0.450^*^	0.293^**^
	**Range**	**2–31**	**1–53**	**7–39**	**1–53**		
**Course**							
**Stationary**	***N***	**3**	**1**	**0**	**1**	0.455^***^	0.289^***^
	**%**	**15**	**2.4**	**0**	**3.3**		
**Progressive**	***N***	**17**	**23**	**6**	**29**		
	**%**	**85**	**54.8**	**100**	**96.7**		

P1, comparison between GG, GC, CC; P2, comparison between GC + CC versus GG; SD, standard deviation

^*^Kruskal Wallis

^**^Mann–Whitney test

^***^Fisher exact test

No significant differences were found in MIF genotypes distribution according to clinical types of the studied vitiligo group.

Within healthy control subjects, vitiligo development was predicted via logistic regression analysis, using age, gender, family history, and MIF genotypes as risk factors. MIF (GC + CC) genotypes were considered as independent predictors for vitiligo development within healthy control subjects (Table 5).

**Table 5:** Regression analysis for prediction of vitiligo development within healthy control subjects

	***p***	**OR**	**95% CI**
Age	**0.549**	**0.995**	**0.981–1.010**
Gender	**0.414**	**1.233**	**0.746–2.038**
Family history	**0.401**	**1.580**	**0.543–4.596**
MIF (GC + CC)	** < 0.001**	**2.626**	**1.559–4.422**

*OR* odds ratio, *CI* confidence interval

Linear regression analysis was done for the prediction of higher disease extent as a sign of disease severity within vitiligo patients, using age, gender, family history, onset, course, and MIF genotypes as risk factors. MIF (GC + CC) genotypes were considered as independent predictors for severity extent within vitiligo patients (Table 6).

**Table 6:** Regression analysis for prediction of higher disease extent within vitiligo patients

	***β***	***p***
Age	**0.3**	**0.125**
Gender	**5.9**	**0.267**
Onset	**0.2**	**0.262**
Course	**13.3**	**0.160**
Family history	**4.9**	**0.606**
MIF (GC + CC) gene polymorphism	**13.7**	**0.007**

*β* regression coefficient

## Discussion

The exact cause of vitiligo is unknown, but genetic and autoimmune factors have been implicated. In addition, altered cellular environment and impaired melanocyte migration and/or proliferation may all contribute to vitiligo etiopathogenesis in varying proportions [12, 13].

MIF was first discovered to be a lymphokine that could aggregate macrophages at areas of inflammation. It is an immune regulatory cytokine that may have a crucial role in various cutaneous disorders [14].

Macrophages are implicated in the elimination of melanocytes in vitiligo. It has been shown that vitiligo lesions contain macrophages, and perilesional skin also exhibits an increase in macrophage numbers [15, 16]. MIF has several biological effects, including activating macrophages, improving their adherence, phagocytosis, and anti-tumor action. Macrophages themselves are a significant source of MIF. Therefore, the MIF and macrophage loop may partially contribute to the etiology of vitiligo [17].

Very few studies have evaluated the elevated serum levels of MIF in patients of vitiligo [17–21]. However, the impact of MIF gene polymorphism in developing vitiligo relies on only two studies: one in China and the other in Western Mexico [22, 23]. To the best of our knowledge, this is the third study worldwide and the first from Egypt to investigate the association between MIF gene polymorphism (rs755622) with vitiligo susceptibility and severity.

Taking GG genotype and G allele as references. Among MIF (rs755622) GC, CC, (GC + CC) genotypes, and C allele showed significantly higher frequencies in cases when compared to the control group. Also, (GC + CC) showed significantly higher BSA% when compared to GG genotype (*p* = 0.002) among studied patients.

Through the logistic regression analysis conducted for the prediction of vitiligo development within healthy control subjects, using age, gender, and MIF (rs755622) genotypes as risk factors. MIF (GC + CC) gene polymorphism genotypes were considered independent significant predictors for vitiligo development within healthy control subjects (*p* < 0.001). However, the linear regression analysis conducted for the prediction of disease severity in the form of higher BSA% among vitiligo patients, using age, gender, onset, course, and MIF (rs755622) genotypes as risk factors, revealed that MIF (GC + CC) genotypes were considered independent predictors for higher BSA% in vitiligo patients.

Our results agree with Wang et al. [22] who found that MIF-173G/C allele and serum concentrations may be associated with active vitiligo. Similarly, Garcia‐Orozco et al. [23] reported that MIF polymorphisms increase the risk of vitiligo in the western Mexican population, and the serum concentrations of MIF and in situ expression are associated with active vitiligo.

These results are in parallel with the role of MIF gene polymorphism in other autoimmune diseases such as psoriasis, atopic dermatitis, alopecia areata, Behçet’s disease, and pemphigus vulgaris.

Ma et al. [24] revealed a link between the MIF 173 G/C polymorphism and atopic dermatitis, and the CC genotype was considerably more prevalent in the subgroup who had asthma and/or rhinitis.

In addition, Nursal et al. [25] found a significant distinction in genotype distribution between Behçet’s disease patients and healthy subjects. When compared to controls, the genotype CC was predominant in patients (*p* = 0.008). Based on GG + GC versus CC genotypes, a significant correlation was found between patients and healthy controls (*p* = 0.003). There was no statistically significant difference in MIF173GC allele frequencies.

Despite the reported elevation of serum MIF levels in psoriatic patients, Shimizu et al. [26] found decreased epidermal MIF-staining in the same lesions. Hence, it was suggested that MIF may decrease in psoriatic lesions to counter regulate the aberrant epidermal proliferation produced by cytokine dysregulation. Moreover, elevated MIF serum levels were associated with severe clinical symptoms of psoriasis.

Furthermore, serum MIF levels were higher in patients suffering from extensive alopecia areata. MIF-173 C allele polymorphisms carry a higher risk of vulnerability to the expansive types of alopecia areata. Given this, MIF has been involved in the etiopathogenesis of the severe forms of alopecia areata [27].

Namazi et al. [28] revealed that the augmented MIF in active pemphigus vulgaris regions may contribute to the disease initiation through T cell activation and B cell stimulation of autoantibodies production. Also, MIF inhibits the effect of corticosteroids; therefore, MIF antagonists may be particularly effective as steroid-sparing medications for this life-threatening condition.

## Conclusion

Based on the results of our study, we can conclude that MIF could play an important role in the pathophysiology of vitiligo and serve as an indicator of disease severity. Therefore, the inhibition of its action may be considered in future therapies of vitiligo. Further multi-center studies with bigger sample sizes and expanding methodology (e.g., functional studies, protein expression correlations) are recommended.

## Data Availability

The data that support the findings of this study are available from the corresponding author upon reasonable request.
